# Neonatal management of parvovirus B19-induced hydrops fetalis: a case report

**DOI:** 10.3389/fped.2025.1611058

**Published:** 2025-11-11

**Authors:** Marco Colombo, Laura Lorioli, Riccardo Pagani, Luisa Patanè, Maurizio Cheli, Giovanna Mangili

**Affiliations:** 1 Clinical Pediatrics, Department of Molecular Medicine and Development, University of Siena, Azienda Ospedaliero-Universitaria Senese, Siena, Italy; 2 Department of Neonatology, ASST Ospedale Papa Giovanni XXIII, Bergamo, Italy; 3 Department of Pediatrics, Milano-Bicocca University, Fondazione IRCCS Ospedale San Gerardo dei Tintori, Monza, Italy; 4 Department of Obstetrics and Gynecology, ASST Papa Giovanni XXIII, Bergamo, Italy; 5 Department of Pediatric Surgery, ASST Ospedale Papa Giovanni XXIII, Bergamo, Italy

**Keywords:** non-immune hydrops fetalis, neonatology, congenital parvovirus B19 infection, fetal anemia, intrauterine transfusion, peritoneal dialysis

## Abstract

**Background:**

Hydrops fetalis (HF) is a severe fetal condition, and congenital Parvovirus B19 (B19V) infection is a leading cause of the non-immune form (NIHF). The recent European B19V outbreak has had a substantial impact on obstetric and neonatal outcomes, leading to an increase in fetal anemia, NIHF, spontaneous abortions, and intrauterine demise. While prenatal diagnosis and intrauterine interventions are well established, postnatal management of B19V-related NIHF remains challenging and outcomes are often poor.

**Case presentation:**

We report a preterm newborn with B19V-associated NIHF following intrauterine transfusions for severe fetal anemia. At birth, the neonate had generalized edema, ascites, and respiratory compromise. Despite aggressive medical management, the patient developed persistent, refractory edema.

**Intervention:**

Given the failure of conservative therapy, peritoneal dialysis (PD) was initiated as a rescue strategy. PD allowed gradual interstitial fluid removal, microcirculatory recovery, and functional “renal rest” after prolonged pharmacologic stress.

**Outcome:**

The neonate progressively improved, with resolution of edema, normalization of renal function, and successful respiratory weaning. At 12 months of corrected age (CA), neurodevelopmental milestones were appropriate, although ongoing long-term surveillance is warranted.

**Conclusion:**

This case contributes to the limited evidence on postnatal management of B19V-related NIHF. PD may be considered in neonates with persistent, refractory edema despite maximal conventional therapy, with careful individualization of treatment. Further research, including multicenter registries and controlled studies, is needed to define its role within standardized neonatal care protocols.

## Introduction

Hydrops fetalis (HF) is a severe and potentially fatal condition characterized by excessive accumulation of fluid within two or more extravascular compartments (1).

The condition is traditionally classified as immune or non-immune (NIHF), with immune hydrops now rare due to the widespread use of anti-D immunoglobulin. NIHF currently accounts for over 90% of cases and encompasses a broad and heterogeneous spectrum of underlying conditions (2). These include cardiovascular abnormalities, fetal anemia, thoracic malformations, intrauterine infections, and a growing list of genetic disorders. Genetic etiologies comprise aneuploidies, pathogenic copy number variations, and single-gene conditions, and their recognition has increased significantly with the widespread adoption of chromosomal microarray and exome sequencing (2, 3).

Among infectious causes, congenital Parvovirus B19 (B19V) infection is one of the leading contributors to NIHF, accounting for approximately 15% of cases (4). The epidemiology of B19V is characterized by cyclical outbreaks every 3–5 years. Notably, the COVID−19 pandemic and subsequent social restrictions temporarily suppressed viral circulation, resulting in a sharp decline in incidence. Since late 2023, a marked resurgence has been documented (5, 6). Recent European surveys (7, 8) have reported a 3- to 10-fold increase in reported B19V cases compared to pre-pandemic levels, although the magnitude varied across countries. In Italy, a multicenter study confirmed this trend, with B19V IgM positivity among pregnant women rising from ∼2% in 2022 to 46% in the first nine months of 2024, and seropositivity in the general population reaching 33.7% (9). B19V is a ubiquitous human pathogen, primarily transmitted through respiratory droplets and blood (10). Infection is most prevalent in childhood, with seroprevalence rates reaching up to 85% in adults (10). Approximately 40% of pregnant women are seronegative (11), and seroconversion during gestation ranges from 1%–2% in endemic periods to 10% during outbreaks (12). Vertical transmission occurs in 17%–35% of maternal infections, typically within 1–3 weeks after maternal exposure, with the highest risk between 9 and 20 weeks of gestation (10).

The pathogenesis of B19V-associated NIHF is complex and multifactorial. B19V induces severe anemia through destruction of erythroid progenitor cells, mediated by its interaction with the P antigen (12, 13). Additionally, it can cause cardiomyopathy via direct cardiomyocyte damage and increase vascular permeability through endothelial infection (14, 15). These mechanisms, particularly high-output cardiac failure secondary to severe anemia, combined with increased capillary permeability, drive fluid accumulation in extravascular compartments, leading to severe and often refractory edema (16). Furthermore, B19V has been associated with thrombocytopenia and acute hepatitis (4, 10), and its tropism for villous trophoblasts can result in placentomegaly (9).

While many congenital B19V infections resolve spontaneously, approximately 5%–10% of cases develop fetal anemia, and 2%–6% progress to NIHF (4, 12). When hydrops occurs, the risk of adverse outcomes rises substantially, with neonatal mortality increasing from around 5% to nearly 50% (11). Furthermore, up to 10% of affected neonates may experience neurodevelopmental delay (12).

We report a preterm neonate with B19V-associated NIHF who developed persistent, refractory postnatal edema despite maximal conventional therapy and was successfully managed with peritoneal dialysis (PD). Although PD has been occasionally used in neonates for various indications, its application in hydrops fetalis remains rare, with only sporadic and outdated publications related to B19V infection (17, 18). Therefore, this case provides novel insight into PD as a postnatal rescue strategy and underscores the importance of standardized, multidisciplinary approaches involving neonatology, cardiology, nephrology, orthopedics, and infectious diseases specialists.

## Case report

### Prenatal course

A 38-year-old woman (G2P1) was admitted at 27.1 weeks of gestation due to fetal hydrops, characterized by subcutaneous edema and ascites, identified during routine ultrasound. No other fetal anomalies were detected. There was no reported family history of erythema infectiosum or respiratory tract infection in the preceding weeks.

Doppler ultrasound revealed a peak systolic velocity of the middle cerebral artery consistent with severe fetal anemia, with an estimated hemoglobin level of 4 g/dl. Serology (positive IgM and IgG) and real-time PCR (viral load >50,000,000 copies/mL) confirmed acute B19V infection. To address fetal anemia, three intrauterine transfusions (IUT) of packed red blood cells were performed via the intrahepatic portion of the umbilical vein: 85 mL at 27.3 weeks, 20 mL at 29.1 weeks, and 18 mL at 31.1 weeks. Post-third IUT, estimated fetal hemoglobin was 13.6 g/dl with a hematocrit of 40%. Despite successful management of anemia, an urgent cesarean section was performed at 32.5 weeks because of uncontrollable labor.

### Immediate postnatal period

At birth, the neonate presented with generalized edema, ascites, and pleural effusions requiring prompt resuscitation with endotracheal intubation. Birth weight was 2,410 g, decreasing to 2,000 g after ascites drainage. Respiratory compromise was attributed to massive ascites causing acute pulmonary hypoinflation and likely limited prenatal lung development, resulting in pulmonary hypoplasia. Despite drainage, gas exchange remained inadequate, with oxygen requirements progressively increasing and the oxygenation index rising to approximately 20. This necessitated escalation to high-frequency oscillatory ventilation (HFOV) from the first day of life (DOL), followed by initiation of inhaled nitric oxide (20 ppm) after echocardiographic confirmation of suprasystemic pulmonary hypertension. Gradual improvement in pulmonary pressures allowed the discontinuation of inhaled nitric oxide after 5 days and a transition back to conventional ventilation, although persistent instability precluded extubation. Initial laboratory tests revealed mild anemia (hemoglobin 11.0 g/dl, hematocrit 32%) with normal platelet counts (450,000 /µl). Two red blood cell transfusions were required during the first week of life to maintain adequate neonatal hemoglobin levels, while platelet counts remained consistently normal. Subsequent echocardiographic assessments revealed a patent ductus arteriosus (PDA) with a minimal left-to-right shunt and no hemodynamic impact, for which conservative management was adopted. Meanwhile, refractory edema emerged as the major determinant of ongoing critical status.

### Shock and edema management

**Figure 1:**
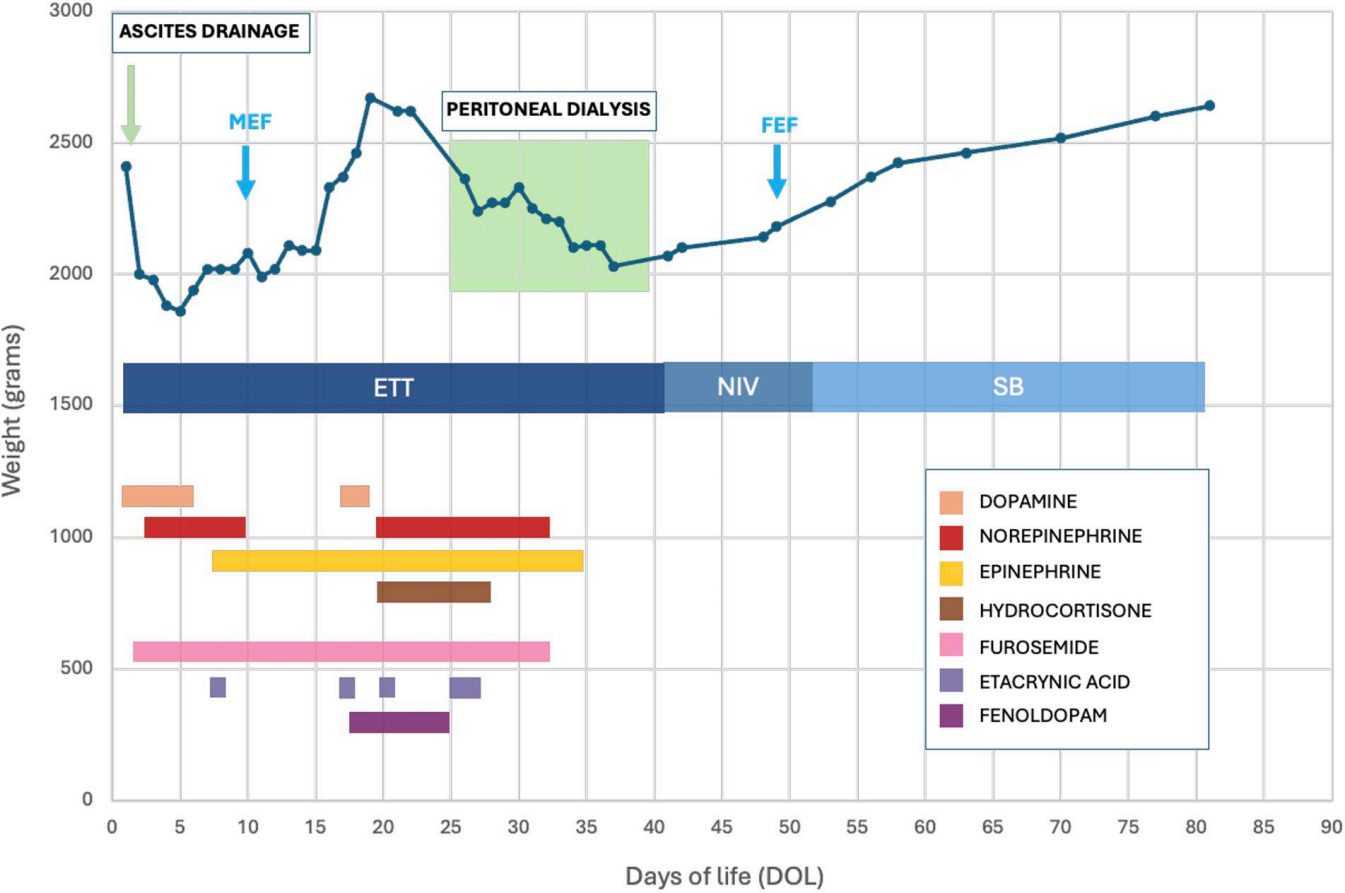
Weight trend and pharmacologic support during hospitalization. Ascites drainage (performed on DOL 0) is indicated by a green arrow, and peritoneal dialysis (performed between DOL 25 and 39) by a green shaded area. The graph also shows respiratory support: ETT (endotracheal intubation), NIV (non-invasive support), and SB (spontaneous breathing). The blue arrows mark the start of MEF (minimal enteral feeding) and the achievement of FEF (full enteral feeding). Pharmacologic therapies included inotropes (dopamine, norepinephrine, epinephrine; *maximum doses*: dopamine 5 µg/kg/min, norepinephrine 0.5 µg/kg/min, epinephrine 0.5 µg/kg/min), corticosteroid (hydrocortisone; *max* 1 mg/kg/day), diuretics (furosemide; *max* 3 mg/kg/day IV boluses or 0.1 mg/kg/h continuous infusion; ethacrynic acid; *max* 1 mg/kg/day), and renal vasodilator (fenoldopam; *max* 0.3 µg/kg/min). Exact regimens varied according to clinical status and are not detailed for brevity.

During the first week, the patient developed severe shock of multifactorial etiology, including both hypovolemic and cardiogenic components. The hypovolemic aspect was primarily driven by increased capillary permeability leading to intravascular volume depletion and interstitial fluid accumulation, whereas the cardiogenic component was related to mild myocardial dysfunction. A multimodal pharmacologic strategy (as detailed in Figure 1) was implemented, including inotropes (epinephrine, norepinephrine, hydrocortisone), diuretics (furosemide, ethacrynic acid, fenoldopam), and osmotic agents (albumin, fresh frozen plasma). Despite these measures, subcutaneous edema persisted and serum creatinine remained elevated (1.15 mg/dl at DOL 25), ultimately prompting initiation of PD as a rescue strategy.

### Initiation and course of peritoneal dialysis

Given the refractoriness of the hydrops and the lack of response to maximal therapy, PD was initiated on DOL 25. PD was considered the most feasible option, enabling gradual interstitial fluid removal, microcirculatory recovery, and functional renal rest after sustained pharmacologic stress. A conservative protocol was used with an iso-osmolar solution (20 mL/kg), initially administered in four cycles per day with 40 min dwell times. This was later reduced to three cycles per day while maintaining a mean negative fluid balance of −18 mL/day. Although net ultrafiltration per cycle was minimal (<5% inflow–outflow difference), PD contributed significantly to clinical recovery.

Throughout treatment, the average spontaneous urine output remained high (6.5 mL/kg/h), allowing for the gradual tapering of diuretics. Objective measures of fluid balance, diuretic therapy, and PD course are summarized in Table 1, which highlights progressive refractory edema before PD and its gradual resolution thereafter. Table 1 presents representative values and overall trends, acknowledging the inherent limitations of precise daily fluid assessment in critically ill neonates.

**Table 1:** Summary of weight trend, fluid balance, renal function, and therapeutic interventions during hospitalization.

Day of life (DOL)	Weight (g)	Fluid balance (mL/day)	Urine output (mL/kg/h)	Diuretic therapy	PD regimen (cycles/day)	PD net balance (mL/day)	Serum creatinine (mg/dl)	Clinical notes
1	2,410–>2,000 (post AD)	NA	1.3	/	/	/	0.91	AD, MV
5	1,880	−20	6.8	FUR bolus (1 mg/kg × 3 /day)	/	/	1.65	Lowest weight
7	2,020	+35 (mean)	5.0	FUR CI (0.1 mg/kg/h)	/	/	1.77	Worsening edema with weight gain
15	2,090		5.0	FUR bolus (1 mg/kg × 2 /day)	/	/	1.11	
25	2,600	−20 (mean)	6.5 (mean)	FUR CI (0.1 mg/kg/h) + FEN	4 cycles/day for 7 d	−18 (mean)	1.15	Initiation of PD
32	2,210			FUR bolus (1 mg/kg × 1 /day)	3 cycles/day for 7 d		0.54	Gradual resolution of edema with weight loss
39	2,070			/	/		0.30	Discontinuation of PD
82	2,640	NA	NA	/	/	/	0.23	Discharge

AD, ascites drainage; CI, continuous infusion; FUR, furosemide; FEN, fenoldopam; MV, mechanical ventilation; NA, not available;/: not administered; PD, peritoneal dialysis.

Daily values are reported when available; mean values refer to the average over the indicated interval.

### Clinical outcome and follow-up

PD was discontinued on DOL 39 following marked clinical improvement: resolution of edema, normalization of renal function (serum creatinine 0.30 mg/dl), and stable electrolytes. No PD-related complications occurred, such as peritonitis, catheter dysfunction, or dialysate leakage. This overall stabilization enabled near-simultaneous withdrawal of supportive therapies, as indicated in Figure 1. From a respiratory perspective, recovery allowed successful extubation on DOL 39, followed by a short course of non-invasive support with high-flow nasal cannula and subsequent stable spontaneous breathing in room air by DOL 46. Echocardiography also documented spontaneous closure of the previously identified PDA.

Nutritional management advanced in parallel with systemic recovery. Minimal enteral feeding (MEF) with maternal milk was initiated on DOL 10, temporarily interrupted during the first three days of PD (DOL 25–28), and then gradually advanced. Full enteral feeding was achieved by DOL 48, exclusively with maternal milk. Throughout fasting and partial feeding phases, individualized parenteral nutrition (PN) was provided, with careful electrolyte monitoring and protein intake adaption to renal function (2.5 g/kg/day during early and dialysis phases, increased to 3 g/kg/day thereafter). Adequate weight gain was maintained throughout hospitalization.

Biochemical monitoring, as detailed in Table 2, revealed early hepatic impairment consistent with acute hepatitis, with elevated aspartate aminotransferase (AST) and alanine aminotransferase (ALT) levels that progressively normalized. B19V DNA quantification revealed a markedly elevated viral load at birth (28,552,387 copies/mL), which declined substantially by DOL 15 (5,301 copies/mL), though complete clearance was not achieved during hospitalization, reflecting persistent, though gradually decreasing, viremia.

**Table 2:** Serial monitoring of renal function, liver enzymes, and parvovirus B19 viremia during hospitalization.

Day of life (DOL)	Serum creatinine (mg/dl)	Blood urea nitrogen (BUN) (mg/dl)	Aspartate aminotransferase (AST) (U/L)	Alanine aminotransferase (ALT) (U/L)	Parvovirus B19 viremia (copies/mL)
1	0.91	39	828	112	28,552,387
5	1.65	129	568	124	NA
7	1.77	231	33	20	7,466,616
15	1.11	303	37	17	5,301
25	1.15	282	34	15	NA
32	0.54	122	65	29	NA
39	0.30	75	84	26	NA
82	0.23	7	85	56	7,228

Neurological monitoring included serial cranial ultrasounds, which demonstrated no major abnormalities. At 44.0 weeks’ postmenstrual age (PMA), brain MRI demonstrated minor punctate changes in the posterior periventricular white matter, interpreted as small sequelae of prematurity without evidence of overt periventricular leukomalacia.

Musculoskeletal screening identified developmental dysplasia of the hip (DDH) with right hip dislocation. An abduction brace was initially applied, but due to insufficient response, surgical correction was performed, resulting in complete resolution.

The neonate was discharged on DOL 82 (equivalent 44.3 weeks’ PMA) in good general condition, with stable growth, normalized laboratory parameters, and appropriate neurodevelopmental progress. At 12 months of corrected age (CA), developmental assessment confirmed age-appropriate psychomotor development.

A structured multidisciplinary follow-up program involving neonatology, cardiology, nephrology, orthopedics, and infectious diseases specialists was planned to monitor long-term outcomes.

## Discussion

Since mid-2023, an unusual surge in B19V infections has been reported across Europe (6, 15), likely reflecting an “immunity gap” created during the COVID-19 pandemic, when circulation of many viral pathogens was suppressed (19). In response, public health advisories have emphasized the increased risk to pregnant women and the potential for severe fetal complications (7, 8). These patterns highlight the need for enhanced maternal screening and timely fetal monitoring, particularly during epidemic periods, to enable earlier diagnosis and intervention and to mitigate the risk of severe outcomes.

Treatment options remain limited. Prenatal interventions are restricted to managing severe fetal anemia (15), while no licensed antivirals exist for preventing or treating B19V infection in pregnancy or the neonatal period (13). Postnatal management of NIHF is particularly challenging when fluid accumulation persists despite maximal conventional therapy.

The pathophysiology of B19V-associated hydrops is multifactorial. The most well-established mechanism is the viral tropism for erythroid progenitors, resulting in aplastic anemia, high-output cardiac failure, and secondary hydrops. Additional mechanisms have also been proposed. B19V DNA and proteins have been detected in endothelial cells of both placental and cardiac tissues, where infection is linked to apoptosis, vascular damage, and release of endothelial microparticles (20, 21). The VP1 unique region of the viral capsid can be internalized by endothelial cells and activate stress pathways even without productive replication (22). Furthermore, antibody-dependent enhancement may facilitate viral uptake into endothelial cells via complement-mediated mechanisms (21). Although evidence in NIHF is limited, endothelial dysfunction and barrier impairment are biologically plausible contributors that warrant further investigation. Understanding these mechanisms could help predict persistent hydrops and guide postnatal management.

From a therapeutic perspective, renal replacement therapies may be considered when conventional management fails (23, 24). In our patient, urine output was preserved and no severe acute kidney injury occurred according to KDIGO criteria (25), yet massive edema persisted, prompting PD as a rescue option. Despite limited supporting evidence (17, 18, 26), PD enabled gradual fluid removal and functional “renal rest” after sustained diuretic exposure. PD was considered the most feasible option in this fragile preterm newborn, whereas alternatives such as continuous renal replacement therapy were unsuitable due to technical limitations and hemodynamic instability.

The evidence base for PD in NIHF is scarce. Early reports from the 1960s–1980s described occasional use (17, 27), but subsequent literature is limited (18, 28). A recent single-center cohort study of 85 neonates confirmed that PD is technically feasible and generally safe, including in hydropic patients, although prognosis largely depends on the underlying disease (29). Our case adds contemporary evidence supporting PD as a rescue option in B19V-related NIHF.

Nutritional management was also crucial given the coexistence of severe hydrops, renal involvement, and the need for strict fluid balance. Current recommendations emphasize the importance of early enteral feeding, individualized PN, and close biochemical monitoring in critically ill neonates (30). In our case, a comprehensive nutritional strategy, combining tailored PN, careful electrolyte monitoring, exclusive maternal milk, and cautious enteral progression, proved essential to sustain growth and recovery while minimizing metabolic complications.

In summary, although our patient achieved reassuring short-term outcomes, long-term neurodevelopmental follow-up remains fundamental to monitor potential late complications (31, 32).

## Conclusion

This case underscores the complexity of B19V-associated NIHF, particularly when hydrops persists despite maximal conventional therapy. While erythroid precursor infection remains the only firmly established pathogenic mechanism, endothelial injury and vascular barrier disruption represent biologically plausible contributors that warrant further investigation. PD may be considered in neonates with B19V-related NIHF who exhibit persistent, refractory edema despite maximal conventional therapy, particularly when urine output is preserved but fluid overload threatens hemodynamic stability. Its application should be carefully individualized until more robust data becomes available. Future research should include mechanistic studies on B19-related endothelial dysfunction, multicenter registries to collect rare clinical experiences, and controlled studies to define the role and safety of PD in NIHF. Ultimately, the management of these infants requires a multidisciplinary approach, integrating cardiorespiratory stabilization, nephrological assessment, tailored nutrition, orthopedic care, and structured follow-up, to optimize both short- and long-term outcomes.

## Data Availability

The original contributions presented in the study are included in the article, further inquiries can be directed to the corresponding author.
